# Local stochastics and ecoclimatic situation shape phytophagous chafer assemblage composition

**DOI:** 10.1002/ece3.10091

**Published:** 2023-05-11

**Authors:** U. G. Sasanka L. Ranasinghe, Jonas Eberle, Suresh P. Benjamin, Dirk Ahrens

**Affiliations:** ^1^ Zoological Research Museum A. Koenig, Bonn Leibniz Institute for the Analysis of Biodiversity Change (LIB) Bonn Germany; ^2^ National Institute of Fundamental Studies Kandy Sri Lanka; ^3^ University of Salzburg Salzburg Austria

**Keywords:** assemblage turnover, body size, chafers, lineages, Sri Lanka

## Abstract

Very little is known about factors determining the assemblage structure of megadiverse polyphagous‐herbivore scarab chafers in the tropics (Coleoptera: Scarabaeidae). Here, we examined the composition of Sri Lankan chafer assemblages and investigated whether it is influenced more by the general ecoclimatic situation, macrohabitat, or indetermined stochastic biotic and abiotic factors of each locality. We also explored the influence of the latter on separate lineages and general body size. Based on dedicated field surveys conducted during the dry and wet seasons, we examined 4847 chafer individuals of 105 species sampled using multiple UV‐light traps in 11 localities covering different forest types and altitudinal zones. Assemblages were assessed for compositional similarity, species diversity, and abundance within four major eco‐spatial partitions: forest types, elevational zones, localities, and macrohabitats. Our results revealed that assemblages were shaped mainly by locality stochastics (i.e., multi‐factor ensemble of all biotic and abiotic environmental conditions at local scale), and to a minor extent by ecoclimatic conditions. Macrohabitat had little effect on the assemblage composition. This was true for the entire chafer assemblage as well as for all single lineages or different body size classes. However, in medium and large species the contrasts between localities were less pronounced, which was not the case for individual lineages of the assemblage. Contrasts of assemblage similarity between localities were much more evident than those for forest types and elevation zones. Significant correlation between species composition and geographic distance was found only for the assemblage of small‐bodied specimens. Seasonal change (dry–wet) in species composition was minor and only measurable in a few localities. The strong turnover between examined localities corroborates with the high degree of endemism in many phytophagous chafers, particularly in Sericini. Connected with their hypothetic poor habitat specificity and polyphagy, this might also explain why so many chafer crop pests in the Asian tropics are endemics.

## Graphical abstract

Synecological analyses on scarab chafer assemblages in 11 localities in Sri Lanka covering different forest types and altitudinal zones revealed that assemblages were shaped mainly by locality stochastics, and to a minor extent by ecoclimatic conditions. Assemblages were assessed for compositional similarity, species diversity, and abundance within four major eco‐spatial partitions: forest types, elevational zones, localities, and macrohabitats. This was true for the entire chafer assemblage as well as for all single lineages or different body size classes.

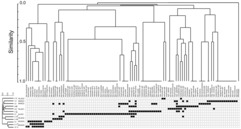

## 1 INTRODUCTION

Analyzing biodiversity patterns is fundamental to understanding the underlying processes and causes of diversification (Holt et al., 2013). In comparison with plants and vertebrates, arthropods are fragmentarily known and lack comprehensive comparative data (Beck & Kitching, 2007; Decaëns, 2010; Nielsen, 2019; Stork et al., 2015). This is particularly true for biodiversity hotspots (Myers et al., 2000). Restricted dispersal capacities of arthropods (Gálvez‐Reyes et al., 2021) and their occurrence in micro‐niches result in fine‐scale high endemism and still unknown patterns (Baselga et al., 2022; Buckley & Jetz, 2007; Daru et al., 2020). Since data on arthropod biodiversity rely often entirely on museum collections, they suffer largely from sampling bias (Echevarría Ramos & Hulshof, 2019; Santos & Quicke, 2011). This is true for even relatively large‐bodied taxa such as phytophagous scarab beetles (Coleoptera: Scarabaeidae).

Diversity patterns of phytophagous beetles are known to be generally linked to species turnover of their host plants (Kemp et al., 2017; Luo et al., 2021; Ødegaard, 2006) and their distribution is correlated with the region and forest type (Yotkham et al., 2021). Other guilds, such as dung‐feeding beetles, respond to shade cover rather than plant species composition. Furthermore, their occurrence and relative abundance vary according as responses to microclimate (light intensity, temperature, and humidity; Davis et al., 2013) or other factors (rainfall, temperature, and host density/diversity) varying from regional to local scale in relation to actual local interactions between organisms and of the organisms with their environment (Tshikae et al., 2013). Such correlation of occurrence and abundance with environmental conditions suggests that a strong role of lineage‐ or species‐specific traits such as dispersal capabilities or body size determines local community composition (Murria et al., 2017). Insect body size is modulated by many climatic factors along species ranges, especially when they are distributed across climatic gradients at large spatial scales (Brehm et al., 2019; Lira et al., 2021; Romero et al., 2016). Changes in body size may affect fertility, lifespan, population dynamics, and species composition (Garcia‐Robledo et al., 2020).

In contrast to most other herbivore insects being rather host‐specific, phytophagous scarab chafers (Coleoptera: Scarabaeidae), with ca 30,000 extant species worldwide, feed unspecifically on leaves of a vast variety of angiosperm plants as adults, on soil humus or roots as larvae (Ritcher, 1958). They have had a very successful follow‐up evolution with angiosperms (Ahrens et al., 2014). However, very little is known about their actual assemblages responding to habitat differences (Eberle et al., 2017), since only few studies have comparatively investigated their quantitative composition (Ahrens et al., 2009; García‐López et al., 2013, 2010), and often these studies include either only a part of the assemblage (Ahrens et al., 2007), and/or consider separate localities rather than habitats (Ahrens et al., 2009; García‐Lopez et al., 2013).

To close this gap, we investigated here patterns of species diversity and turnover in tropical phytophagous chafers in Sri Lanka, a global biodiversity hotspot (Myers et al., 2000) across different forest types, elevation zones, localities, and habitats. We attempt to explore to which extent each of these spatial components determines assemblage composition. In this context, we also assessed their influence on different lineages and the role of body size in shaping species composition. If body size (as proxy of dispersal capability) had an impact on assemblage composition, we would expect contrasting patterns between entities of different spatial scales between smaller and larger species assemblies, also in respect to phylogenetically partitioned assemblages. This way, we expect to elucidate the dynamics of community assembly and differentiation and to explain the high species richness and endemism in tropical chafers.

## 2 MATERIALS AND METHODS

### 2.1 Study area and sampling

Four field expeditions in Sri Lanka were conducted during 2019 and 2020 (February–March/October–November and June–July/November–December, respectively) during dry and rainy seasons. Specimens were sampled using six UV‐light traps per locality site (Ranasinghe et al., 2020) in 11 localities covering different four forest types, as defined in a sense of entities of potential natural vegetation (Dittus, 1977; Holmes, 1956; Legg & Jewell, 1995; Perera, 2001), and five altitudinal zones each covering an interval of 500 m (Figure S1). Sites were in evergreen wet lowland forests (below 500 m: L1, L8, L9; or above 500 m: L12, L13, L14), evergreen dry lowland forest (L3), submontane forests (L2, L4), or montane forests (L5, L11; Figure S1, Table S1). Traps were placed in each locality at different sampling sites (i.e., macrohabitats) at approximately 2 m above ground (Table S1). They were positioned at the same location in each campaign for 2–3 consecutive days and operated from 6 to 11 p.m. All traps (traps A‐F) were separated by a distance of at least 100–500 m, to not influence each other. Beetles were trapped in a sampling container with preservation liquid (96% ethanol; for trap design, Figure S2; see also Ranasinghe et al., 2020; Ranasinghe, Eberle, Athukorala, et al., 2022). Specimens were preserved in 96% ethanol. In total, we performed 10–12 trapping events per expedition/site (i.e., 1 trapping event = 1 night per 1 trap), resulting in 60–72 trapping events in each location.

Phytophagous chafers (Coleoptera: Scarabaeidae) in Sri Lanka include Dynastinae, Melolonthinae, and Rutelinae. Specimens were identified to morphospecies based on external and genital morphology; some being subsequently examined by a taxonomic specialist of the group. The majority of herbivore scarabs is night active, only a few exceptional and rare diurnal species exist in Sri Lanka (e.g., *Periserica*; at maximum 1–2 species per locality). Other pleurostict diurnal species comprise mainly floricolous, pollen‐feeding species (few *Popillia* (Rutelinae) species and Cetoniinae). Specimens are deposited in the Zoological Research Museum A. Koenig, Bonn (ZFMK) and in the National Institute of Fundamental Studies, Kandy, Sri Lanka (NIFS).

### 2.2 Assemblage characterization

Species richness and abundance were assessed by the mean number of species or individuals, respectively, per trapping event and site (i.e., total number of specimens per species of a particular trap divided by number of used traps). Thus, abundances were corrected for sampling biases due to trap failures because of weather or technical problems. Species presence–absence data were used for the assessment of species composition and assemblage similarity. Species accumulation curves were plotted for each trap with the cumulative number of recorded species vs. number of cumulative trapping events to assess sampling adequacy and comparability of the results. Species' completeness in each sampling locality was assessed by the number of observed species in respect to the number of species predicted by the Chao1 richness estimator, that is, the total number of species in each locality with lower and upper limits (Chao & Lee, 1992; Zou & Axmacher, 2021). Sampling data (i.e., specimens per trap) were pooled for each trap from all four sampling campaigns (2019 I, 2019 II, 2020 I, and 2020 II) for total assemblage analyses. A two‐way cluster analysis (species vs. locality) was performed based on presence–absence data using the Jaccard similarity index (Jaccard, 1912) in PAST v. 3.25 (Hammer et al., 2001).

The alpha diversity was measured using Shannon index, Simpson index, and Evenness for each locality (Hill, 1973; Jost, 2006; Shannon, 1948; Simpson, 1949) being calculated in PAST v. 3.25. Approximate confidence intervals for all these indices were computed with a bootstrap procedure (number of random samples [default 9999] with 95% confidence interval). Furthermore, Hill numbers (*q* = 1 and *q* = 2) were calculated using the “Diversity Excel Template” as implemented in the AHP‐OS package (Goepel, 2018).

To examine hierarchically the overall similarities between the sampling localities and to identify species with similar occurrences, we performed a two‐way cluster analysis (dendrogram; UPGMA) based on (a) the species present/absent in sampling localities and (b) the presence/absence of locality records for species (Jaccard similarity index).

### 2.3 Assemblage assessment partitioned by body size and lineages

Interspecific differences in body size may reflect divergences in species ecology and behavior (Eberle et al., 2014; Inward et al., 2011; Lira et al., 2021). Thus, size‐related differences in assemblage composition across different spatial scales may provide insight to the causalities of these patterns. Body size groupings were based on initial analyses which revealed no gaps in size distribution. Therefore, we have chosen arbitrarily (just) three classes to have sufficient within‐group distribution. Therefore, assemblages were analyzed according to these three body size groupings which reflect in large part lineage‐related size distribution (genus level): (1) smaller 7 mm; (2) medium 7–15 mm; and (3) larger 15 mm. However, most species were part of the small and medium size class. The respective total body length was calculated using the sum of pronotal and elytral length (PL + EL). The mean total body length of a species was determined by taking the mean value of three to five individuals of the same species. In order to also consider an even distribution of species over size classes, we tested for an alternative size partitioning scenario, using a 33% percentile of the total distribution (i.e., subdividing the assemblages also into three groups; see also Figure S4). Finally, assemblage composition analyses were partitioned according to membership of phylogenetic lineages (following McKenna et al., 2019): Dynastinae, Rutelinae, Melolonthinae (excluding Sericini), and Sericini to explore also phylogenetic patterns of differences in assemblage composition (Smith et al., 2021). The latter subdivision of Melolonthinae was investigated due to its paraphyly under current classification (Bouchard et al., 2011), which has been shown in several molecular phylogenies (e.g., Ahrens et al., 2014; Eberle et al., 2019; McKenna et al., 2019).

### 2.4 Spatial turnover analysis

Non‐metric multidimensional scaling analyses (NMDS) based on presence–absence data using the Jaccard similarity index were performed for four major spatial components (i.e., forest types, elevational zones, localities, and habitats). For this purpose, each single trap was assigned for a particular spatial component (Table S1). Forest types included four entities: (a) evergreen wet lowland forests, (b) evergreen dry lowland forests, (c) submontane forests, and (d) montane forests. Elevation had five units: EZ1: 0–500 m, EZ2: 501–1000 m, EZ3: 1001–1500 m, EZ4: 1501–2000 m, and EZ5: 2001–2500 m. The entity ‘locality’ included the 11 individual sampling localities. Habitat comprised seven types: abandoned plantation, grassland, rock outcrop, hilltop, forest edges, central forest, and disturbed forest (here, the habitat includes smaller‐scale “microhabitats” of soil‐dwelling saprophagous larvae, and herbivorous adults). NMDS ordination was performed on the full data set. Entities of spatial components (i.e., forest types, elevational zones, localities, and habitats) were subsequently mapped on the ordination results. Spatial turnover analysis as well as a regression between qualitative species composition similarity and geographic distances of sampling sites were performed for the full assemblage and for assemblages partitioned by body size and phylogenetic lineage membership (see above).

### 2.5 Seasonal turnover analysis

We assessed seasonal turnover for single traps and localities using NMDS ordinations based on Jaccard indices from species presence–absence data. The turnover of species composition in time was also evaluated for the localities through ANOVA and Kruskal–Wallis tests as implemented in PAST. Finally, we compared also seasonal turnover for lineage‐ and body‐size‐partitioned assemblage data.

## 3 RESULTS

A total of 4847 specimens of 105 chafer species belonging to Rutelinae, Melolonthinae, and Dynastinae were recorded (Table S2). Species richness estimators suggested >89% of total species inventory had been captured. While 82% of the individual locality assemblages showed more than 84% of sampling completeness (in terms of species composition), in two cases sampling completeness was, with <50%, quite low (L9, L14; Table S3). Species accumulation curves for individual localities showed species saturation and about 80% of its species have been captured in less than half of the total trapping events (before 34th trapping event; Figure S3). Similarly, species accumulation curves for individual traps showed that about 80% or slightly more of the expected species has been captured before half of the total trapping events.

**FIGURE 1:**
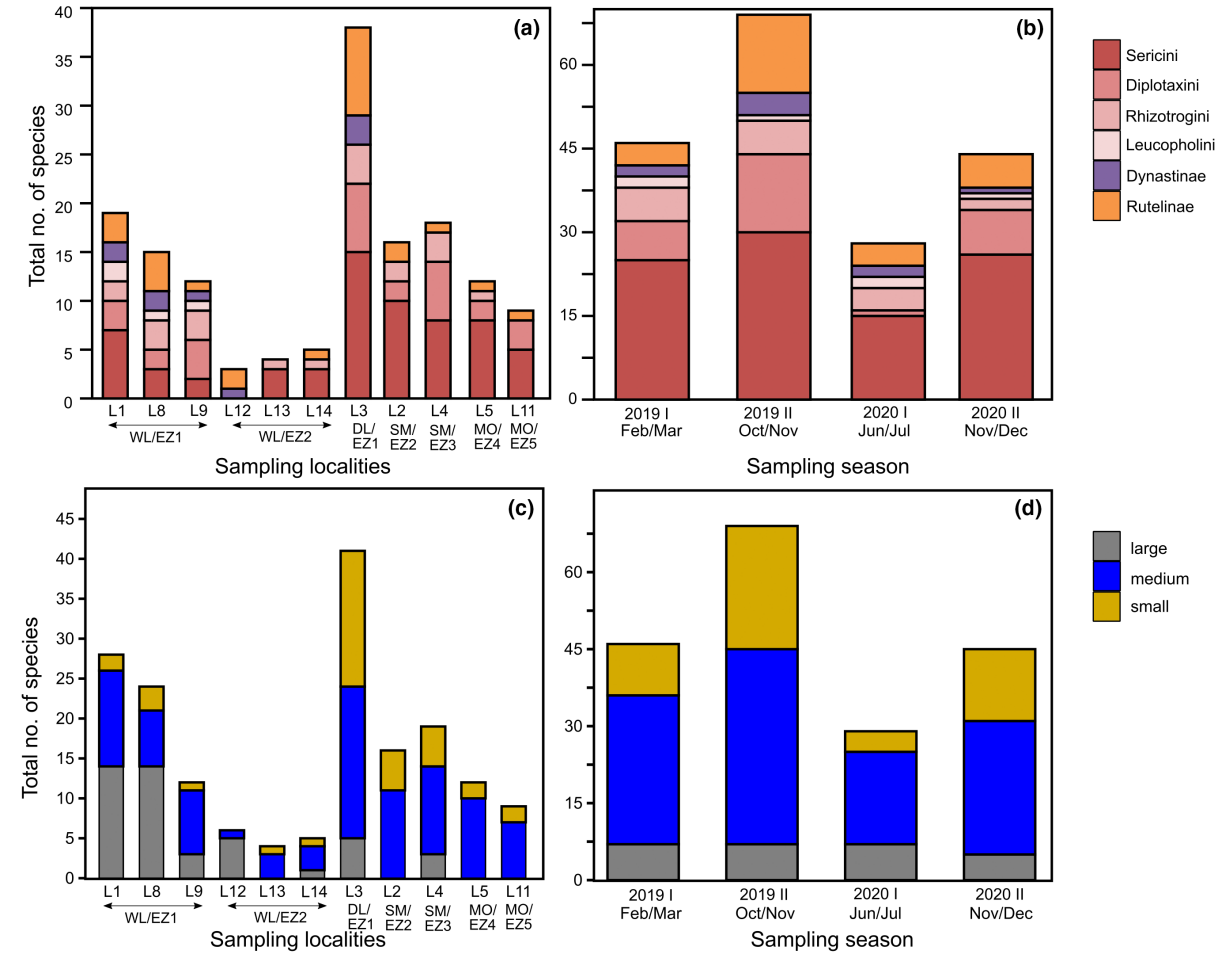
Total number of species (species richness) in different locations and in four field campaigns; (a and b) based on subfamily level/separate lineages; (c and d) assemblage sorted for body size.

**FIGURE 2:**
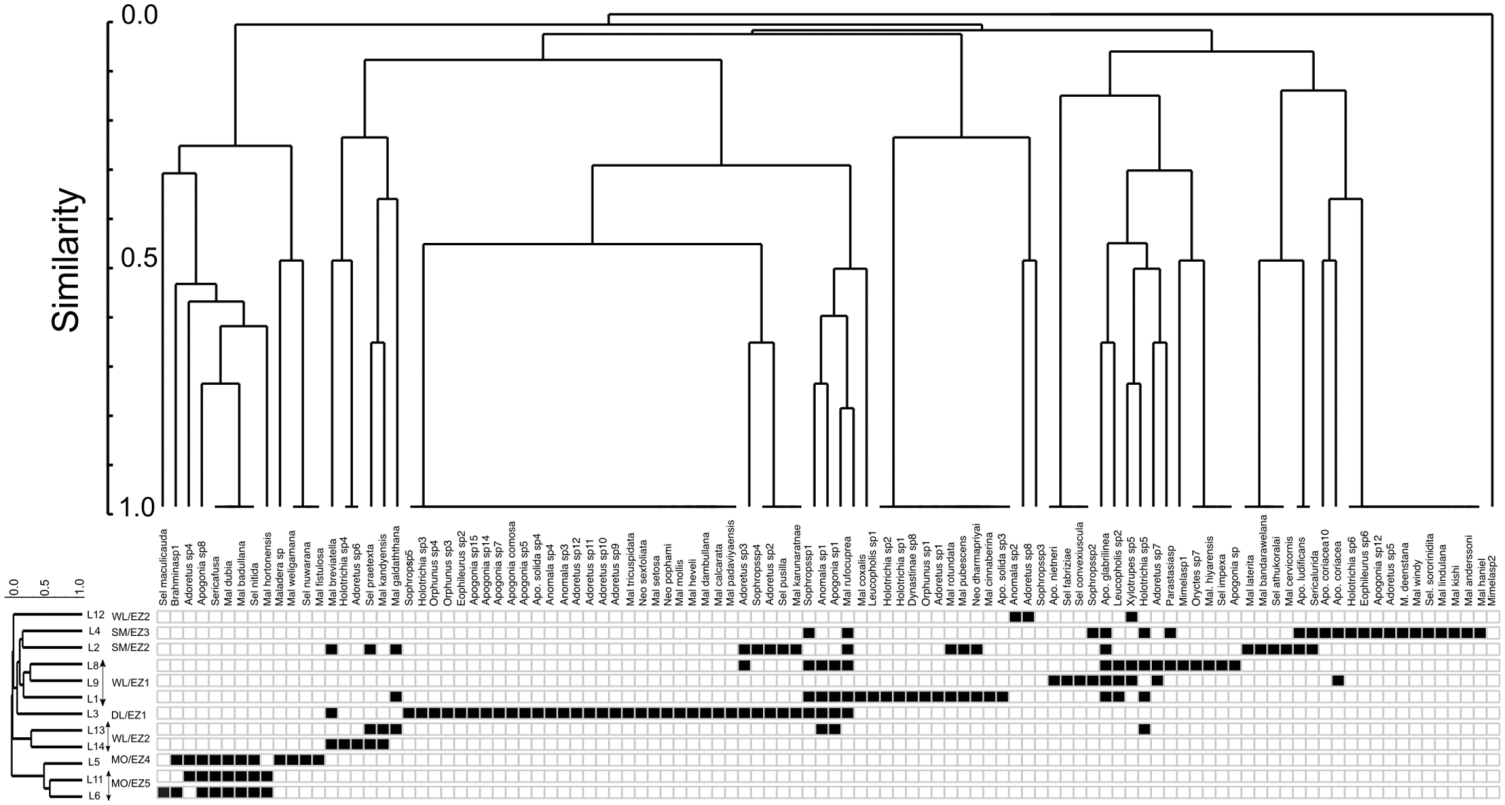
Summary of species presence (black square)/absence data (white square) and resulting similarity of study sites (left part) and species occurrences (upper part) based on a two‐way cluster analysis (dendrogram; UPGMA) based on the species present/absent in sampling localities (Jaccard similarity index).

Melolonthinae was the most speciose subfamily (*n* = 79), with the highest number of recorded individuals (*n* = 2504). Dynastinae had the lowest number of species (*n* = 8) and individuals (*n* = 38). For Rutelinae, we recorded 18 species in 531 exemplars. Among the Melolonthinae, Sericini was the most speciose tribe accounting 44.7% of all species (Figure 1). Many species were geographically restricted, 67 species out of 105 (64% of total assemblage) were found exclusively at just one site. L3 showed the highest alpha diversity and L13 the lowest which was reflected by all diversity indices (Table S3). Shannon index and Hill numbers (*q* = 1, *q* = 2) produced quite similar results. These patterns are also reflected by the results of the two‐way cluster analysis, one for the species occurring in different localities, and another for the different localities in which certain species are present (Figure 2), which linked faunal similarity with similar species occurrence patterns.

### 3.1 Spatial turnover

**FIGURE 3:**
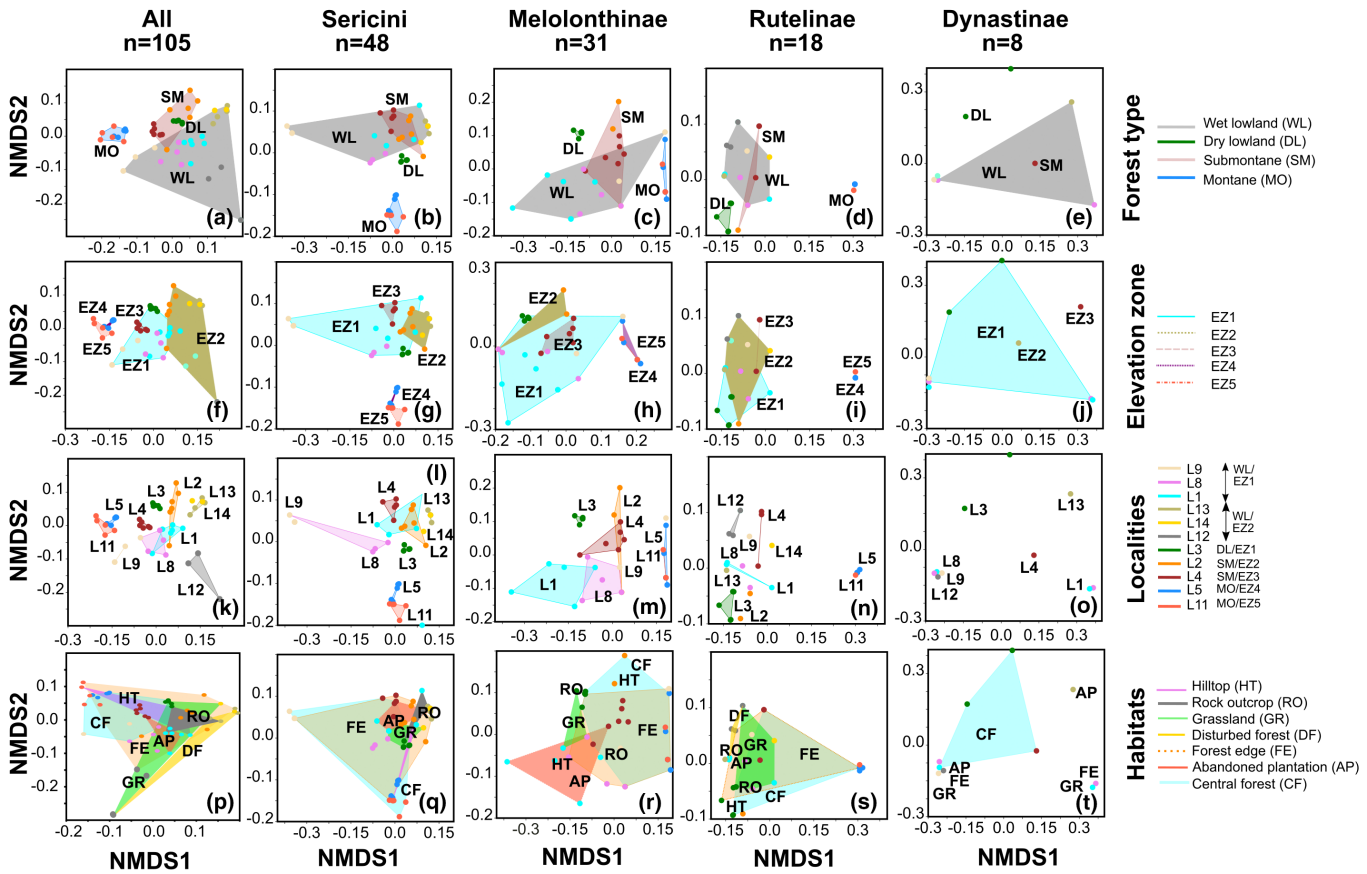
NMDS analyses of assemblages from single trapping events separated by lineages and different spatial and eco‐spatial partitions; forest types (a–e), elevation zones (f–j), localities (k–o), and habitats (p–t). Partitions are enclosed by convex hulls. Multiple traps from one locality have the same color and colors correspond to Figure S1.

Ordination analysis on species presence/absence data (NMDS) of the full chafer assemblage generally showed different patterns for the different eco‐spatial components (Figure 3a,f,k,p). The largest overlap of entity clusters was observed for the macrohabitats. Overlap in forest types, elevation zones, and localities were limited to a few entities; most entities were well‐separated. The distances between the entity clusters were almost similar within the same spatial component. Similar patterns were also observed for separate lineages; however, differences between the single entities (e.g., elevation zones or forest types) were less pronounced with slightly larger overlaps. For Dynastinae, patterns were not well pronounced due to low sample representation (Figure 3). Species composition of montane forest localities (L5, L11) resulted generally more similar to each other compared with the rest (Figure 3), while assemblages of dry lowland forest were dissimilar from the remainder for single lineages but not for the entire assemblage.

**FIGURE 4:**
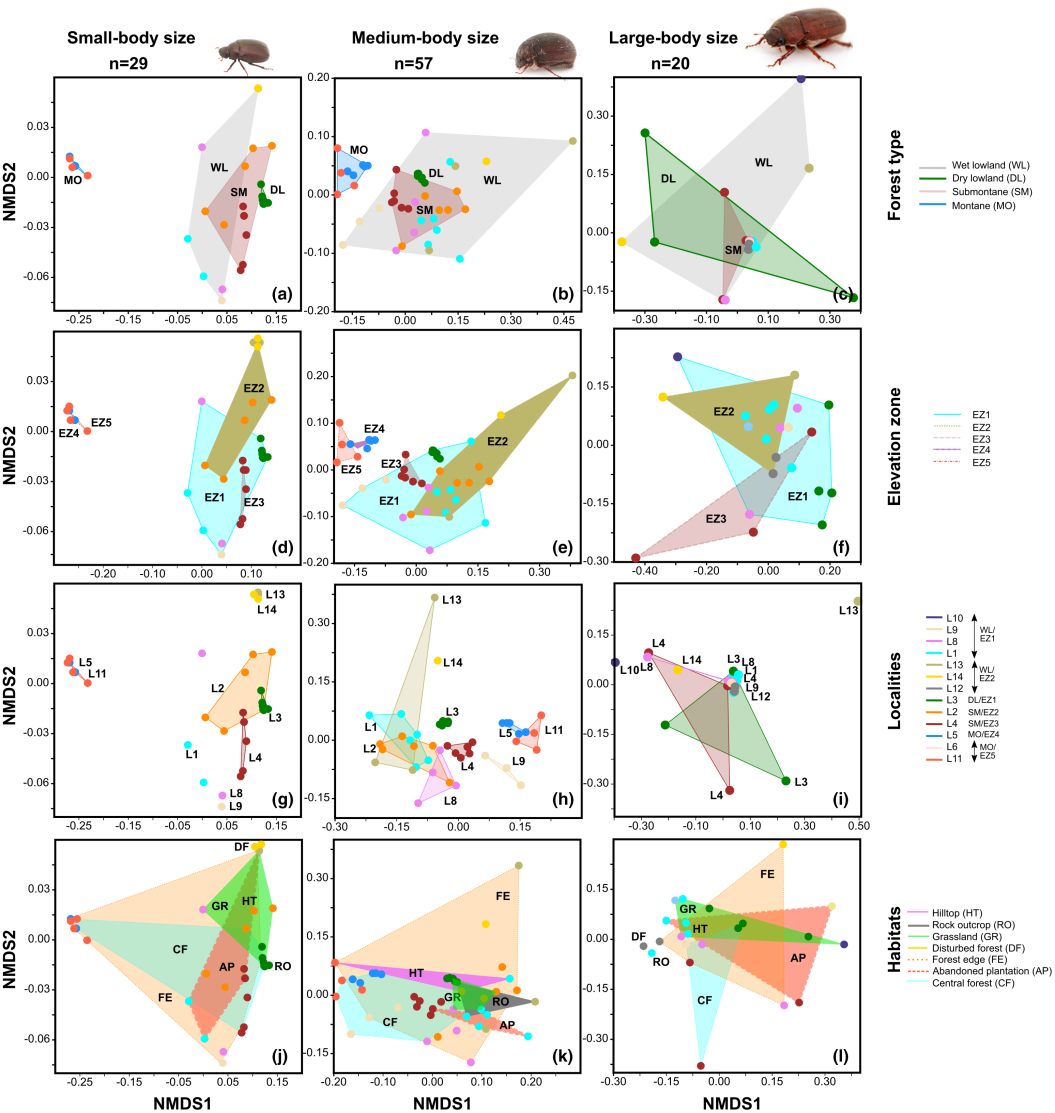
NMDS analyses assemblages separated by body size classes and different spatial and eco‐spatial partitions; forest types (a–c), elevation zones (d–f), localities (g–i), and habitats (j–l). Partitions are enclosed by convex hulls. Multiple traps from one locality have the same color and colors correspond to Figure S1.

NMDS on Jaccard indices from species presence–absence data for the three different body size classes showed similar overall patterns, for both size partition approaches: large overlap for all partitions in macrohabitats, and moderate to clear distinction for ecoclimatic zones (elevation and forest type) and localities. Small and medium‐sized assemblages showed somewhat contrasting patterns for assemblages of large‐bodied specimens for forest types, elevation zones, and localities (Figure 4, Figure S4). Again, eco‐spatial entities (e.g., elevation zones, or forest types) in partitioned analyses were less different than for the full assemblage data (Figure 4, Figure S4).

**FIGURE 5:**
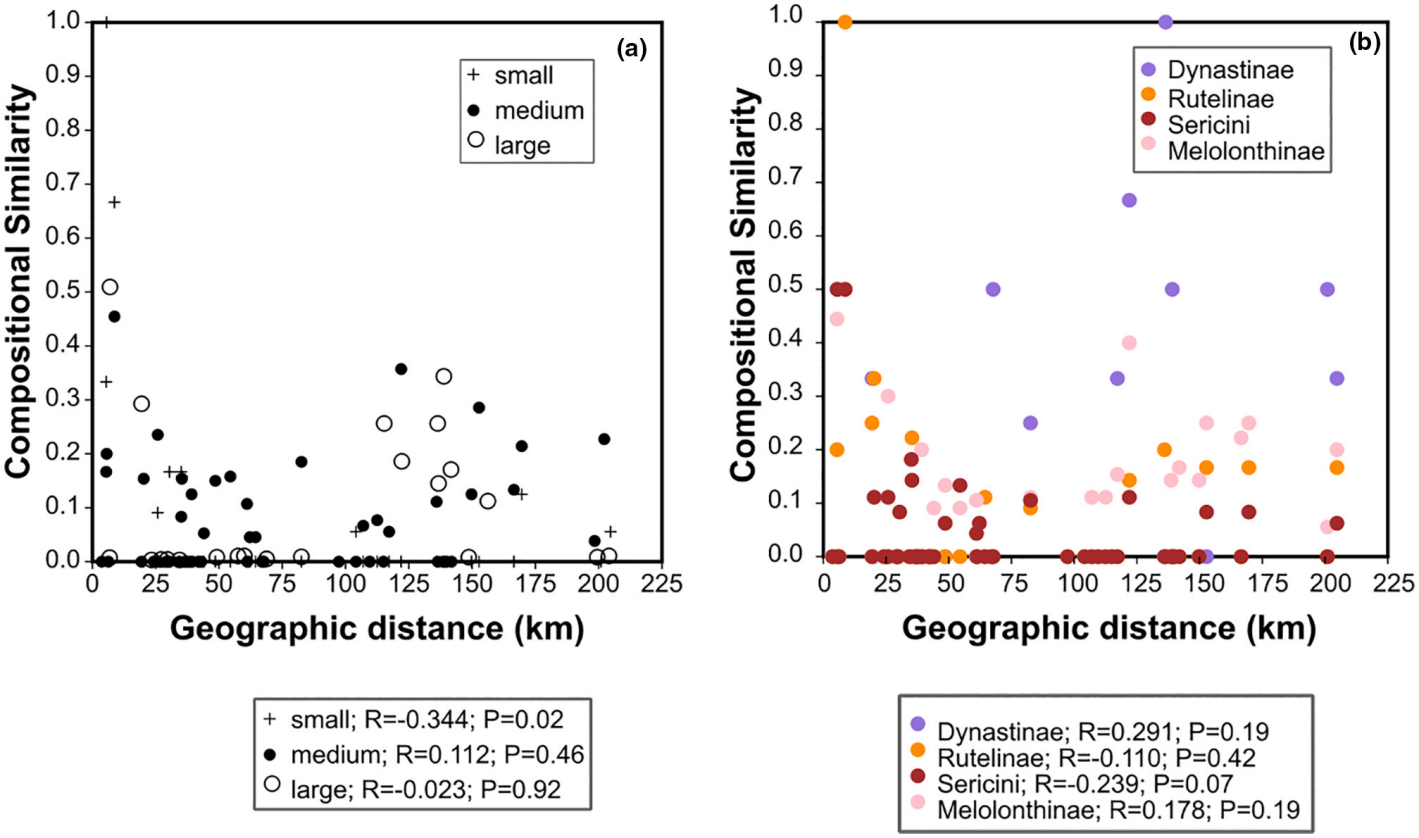
Correlation between species compositional similarity (and pairwise geographic distance). (a) Assemblage sorted for body size; (b) assemblage sorted for lineages.

A linear correlation analysis showed no significant correlation (*r* = −.029, *p* = .831) between compositional similarity and geographic distance among localities (Figure 5). We also tested for this correlation for the assemblages partitioned by body size and lineages (Table S4); a significant relationship between species composition similarity and geographic distance was found only for the assemblage of small‐bodied specimens (*r* = −.344, *p* = .02).

### 3.2 Seasonal turnover

**FIGURE 6:**
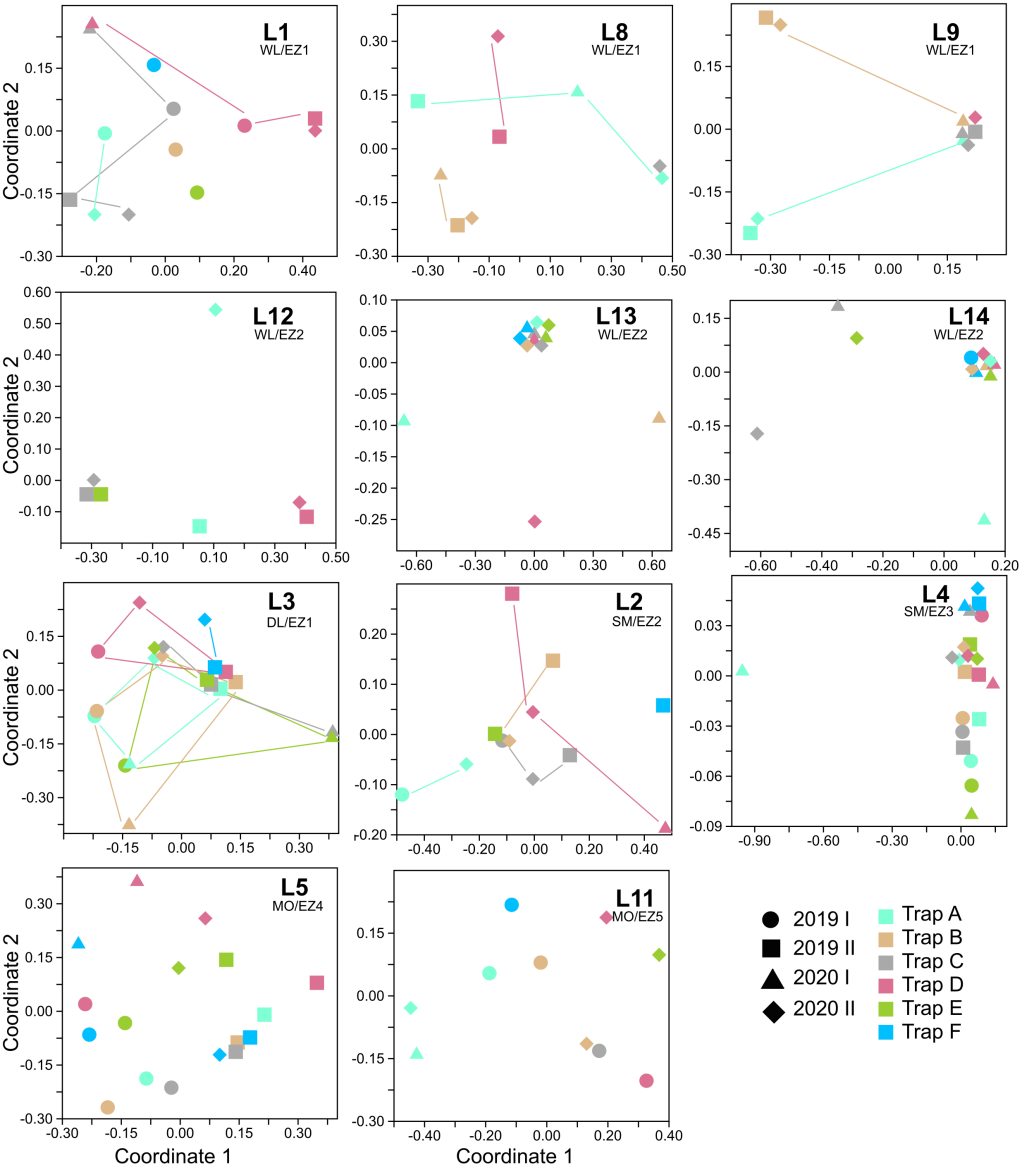
NMDS analyses of assemblages from single trapping events separated by sampling locality in the course of four field campaigns (2019 I, 2019 II, 2020 I, and 2020 II).

Species number and abundance varied significantly between the four field campaigns (ANOVA, *p* < .01; Figure 1b). Patterns of species turnover among single traps between the sampling campaigns were not homogeneous for different localities. Some localities showed very little difference in species composition between all campaigns, some had generally strong differences between all campaigns, and in some cases, only one or two campaigns differed compared with others (Figure 6). For data pooled for locality and field campaign, seasonal species turnover of localities varied between 19% and 61% (Table S5). Kruskal–Wallis test for individual localities showed that L1, L2, L3, and L9 had a significant seasonal species turnover, while other localities did not show any significant differences in seasonal composition (Table S5). Among our four field campaigns, February (2019 I) and December (2020 II) campaigns showed the highest faunal similarity (i.e., 49.2%) and lowest similarity (17%) was found between campaigns of October (2019 II) and June (2020 I; Table S6). Faunal similarity among campaigns varied for lineage and body size partitioned assemblage data, which showed higher similarity for Melolonthinae and Sericini as well as small‐sized specimens compared with the complete assemblage, in all other less faunal similarity compared with the latter (Table S6).

## 4 DISCUSSION

We investigated here for the first time components determining the chafer assemblage composition, comparing the impact of ecoclimatic influences with macrohabitat and locality stochastics on the similarity of investigated entities. Locality stochastics represent a not further investigated multi‐factor ensemble that includes all biotic and abiotic environmental conditions at local scale such as macrohabitat, biogeography, edaphic conditions, land use, predation, local climate, rainfall, and radiation. We also explored the patterns of lineage membership and body size resulting from assemblage composition across larger scale entities (forest type, elevation) versus smaller‐scale entities (localities and macrohabitats).

The comparison of chafer assemblage composition at different eco‐spatial scales revealed that assemblages were shaped mainly by locality stochastics, to minor extent to the ecoclimatic conditions, and not by macrohabitat. This was true for the entire chafer assemblage, as well as single lineages or different body size classes. NMDS plots of faunal similarity showed the largest overlap among macrohabitat entities. In contrast to that, overlap for clusters of forest types, elevation zones, and localities was limited. However, contrasts between localities were less pronounced in medium‐sized and large specimens.

Investigated macrohabitats were quite different (e.g., forest, grassland, and abandoned plantations). They are known to provide multiple niches (Bosc et al., 2019) for chafer species; however, only a few species were recorded that were specific to these habitats. Most species and resulting assemblages sorted by locality rather than by macrohabitat. This could be partly explained by the trapping method (light traps) used, as fully winged chafer beetles may be attracted from other habitats over certain distances within the same locality. However, the fact that we found no correlation between species composition (for total assemblage) and geographic distance (Figure 5), even for adjacent localities situated in the same forest type also in the same mountain range (e.g., L2, L4), may indicate either that species generally might tend to disperse also over moderate‐to‐longer distances or that species disperse very little. Limited dispersal is supported further by molecular evidence (Ranasinghe et al., in review; Ranasinghe, Eberle, Thormann, et al., 2022), since different, the same here investigated localities shared almost no haplotypes. This latter conclusion would be not surprising as previous studies have also shown high turnover rates of assemblage composition at higher elevations independently from geographical distance (García‐López et al., 2010). However, the resulting significant correlation for the assemblage of small‐bodied specimens, which is definitively linked to their limited dispersal capacity and mirrored by their higher endemism (Fabrizi & Ahrens, 2014), might indicate that lacking significance on our study might be a result of an insufficient number of samples and species. Larger species were generally less common and are also less represented in higher altitudes. Influence from paleogeographical and biogeographical factors should also be considered in this context (Kemp et al., 2017) as several sampling localities are situated in the central highlands within complex mountain systems (escarpments, ridges, or peaks), which can act as geographical barriers. The latter can particularly triggered geography‐driven speciation, as shown by diversification of Sericini in Asian mountains (Ahrens, 2007; Eberle et al., 2016).

Some of the divergent composition patterns retrieved for the full assemblage (Figure 3a,f), which are in turn not encountered for any of the single lineages, reveal that occurrences of entire lineage members may also impact on the apparent differentiation (e.g., wet lowland forest vs. submontane forest, EZ 1 vs. EZ 2). The latter case is caused by the more poorly sampling/ absence of larger‐bodied species (e.g., Dynastinae), in higher elevations, since low temperatures obviously might not favor larger species with long larval development (Danks, 1992). In fact, even in mountain ranges with larger amplitudes of elevations, the altitudinal differentiation of the fauna in phytophagous chafers is rather poor (Ahrens, 2004) compared with other insects (Mani, 1968).

The strong turnover for localities is in line with the rather high degree of endemism in many phytophagous scarabs (Ahrens & Fabrizi, 2016), despite their considerable size. Their assemblage patterns across local spatial scales can be explained not only by poor dispersal capacities, but also by short emergence times compared with the length of their life span: Their root‐feeding, endogenous larvae do not disperse. Their emergence during early night time often falls together with heavy monsoon rains which narrows down the time window for potential dispersal flights. The poor habitat specificity might also explain, why so many chafer pest species in the Asian tropics are local endemics. Given their independency of the crop plant due to their polyphagous herbivory, original forest species may often use cultivated open lands (Ahrens et al., 2009). They often remain unidentified when the taxonomy of the group is poorly studied (Ahrens & Fabrizi, 2016).

Other lineages composed of larger species, such as Dynastinae, have greater dispersal ability compared with smaller Rutelinae and Melolonthinae (García‐Lopez et al., 2013), and this has an impact on the faunal divergence pattern of assemblages as revealed by pronounced larger cluster overlaps across different spatial scales.

The literature record for the correlation of size vs dispersal capacity for chafers is rather poor and may also strongly depend on other factors such as topology and emergence duration. We know many larger chafers species have also larger ranges, while certain lineages such as Sericini have not difference in endemism in regard of size. Body size can be a plausible proxy for dispersal capability in chafers but remains currently a rather descriptive element to be explored further, for which this study could be best stimulus.

Seasonality and weather fluctuation may strongly impact the expressed patterns of assemblage composition in ecofaunistic analyses (de Oliveira et al., 2021). In tropical climate, rainy seasons and dry seasons are alternating in shorter intervals with quite constant temperature and humidity throughout the year and food resources being continuously available. Thus, minor fluctuations to species' presence and numbers may occur even in the tropical ecosystems. Many of our localities (except L1‐L3, L9) did not show a significant seasonal species turnover, while those which did experience generally stronger dry–wet fluctuations than other localities according to their position in the island.

In the final conclusion, we need to remember that at local level all ecological, climatical, and spatial components sum up in their effect increasing the complexity of influences on the assemblages. This points the way for future, more detailed studies, in which localities of similar eco‐spatial situations shall be addressed. Yet, since phytophagous chafers are common pests for many tropical crops, damage can often also be caused by a multispecies autochthonous community with endemic species (Ahrens et al., 2009). For this reason, our insights for factors determining the assemblages of natural chafer assemblages are crucial primer for the further understanding of the evolution of this group but also for being able to manage more sustainably chafer pests. Therefore, further and more robust knowledge on assemblage ecology is desirable, which can be achieved only by more rigor, more sampling, an understanding of legislation, and conservation management for the need to sample and kill multitudes of insects to study ecology appropriately, and a minimum of funding.

## AUTHOR CONTRIBUTIONS

**U. G. Sasanka L. Ranasinghe:** Conceptualization (equal); data curation (equal); formal analysis (equal); funding acquisition (equal); investigation (equal); methodology (equal); project administration (equal); validation (equal); visualization (equal); writing – original draft (equal); writing – review and editing (equal). **Jonas Eberle:** Conceptualization (equal); methodology (equal); supervision (equal); validation (equal); writing – original draft (equal); writing – review and editing (equal). **Suresh P. Benjamin:** Funding acquisition (equal); resources (equal); supervision (equal); writing – original draft (equal); writing – review and editing (equal). **Dirk Ahrens:** Conceptualization (equal); data curation (equal); funding acquisition (equal); investigation (equal); methodology (equal); project administration (equal); resources (equal); supervision (equal); validation (equal); visualization (equal); writing – original draft (equal); writing – review and editing (equal).

## CONFLICT OF INTEREST STATEMENT

We have no conflicts of interest to declare.

## Supporting information


Data S1

Data S2

## Data Availability

All supporting data are made freely available in Dryad and as Supporting Information.
