# Supplementary material for: Local stochastics and ecoclimatic situation shape phytophagous chafer assemblage composition
Source: Ecol Evol. 2023 May 11;13(5):e10091. doi: 10.1002/ece3.10091 (PMC10175718; doi:10.1002/ece3.10091)
Supplement: Supplementary file 1 — Data S1 [file ECE3-13-e10091-s002.pdf]

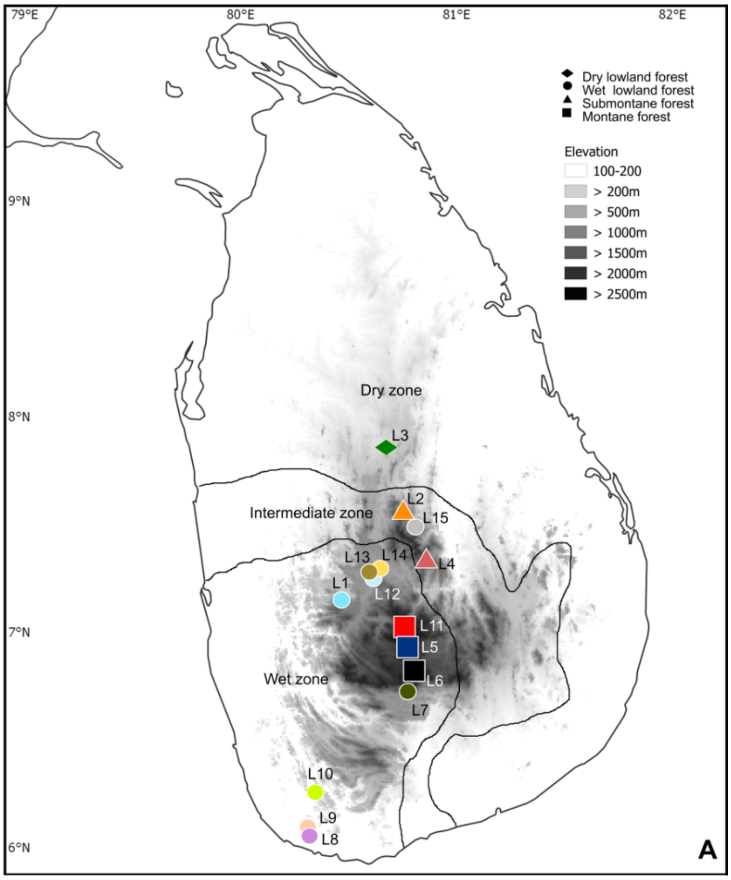

Figure S1: Map of Sri Lanka showing sampling sites. L1: Aranayake; L2: Riverston; L3: NIFS Arboretum; L4: Deenston; L5: NuwaraEliya; L6: Horton Plains; L7: Belihuloya; L8: Hiyare; L9: Kottawa; L10: Kanneliya; L11: Piduruthalagala; L12: Uda Peradeniya; L13: Gannoruwa; L14: Udawattakele. Symbols represent different forest types.

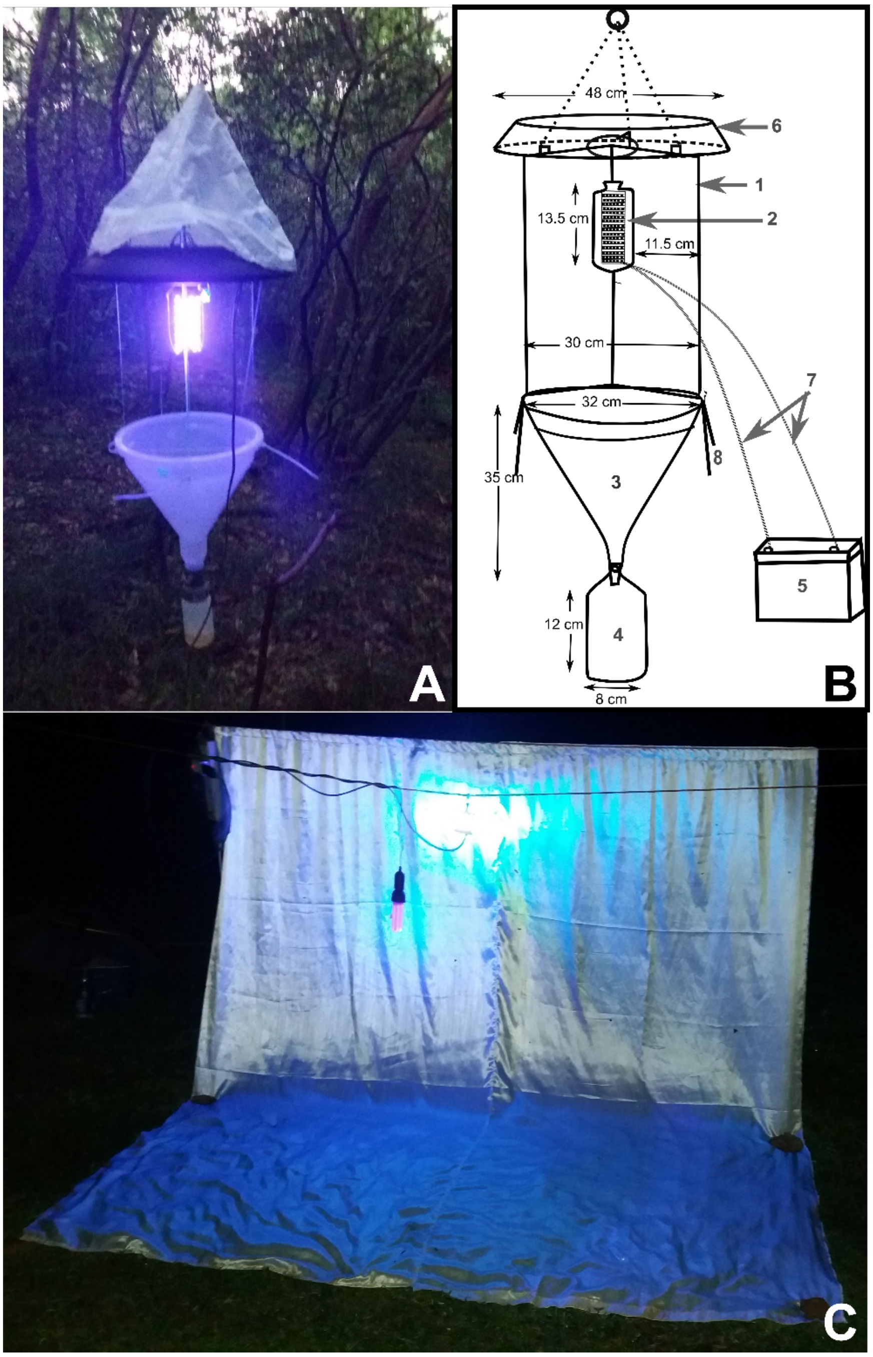

Figure S2: A. Light trap used in the field for this quantitative survey of the chafer fauna. **B.** Sketch of the light trap including 1: transparent polystyrene plates, 2: light source, 3: funnel, 4: container, 5: battery, 6: plastic lid, 7: battery cable, 8: plastic strip cable zip ties. **C**. Sampling (for purposes of taxonomic investigation and DNA barcoding) was completed by manual collecting from a white sheet illuminated with UV light in the field, however, these latter data were not included in this study.

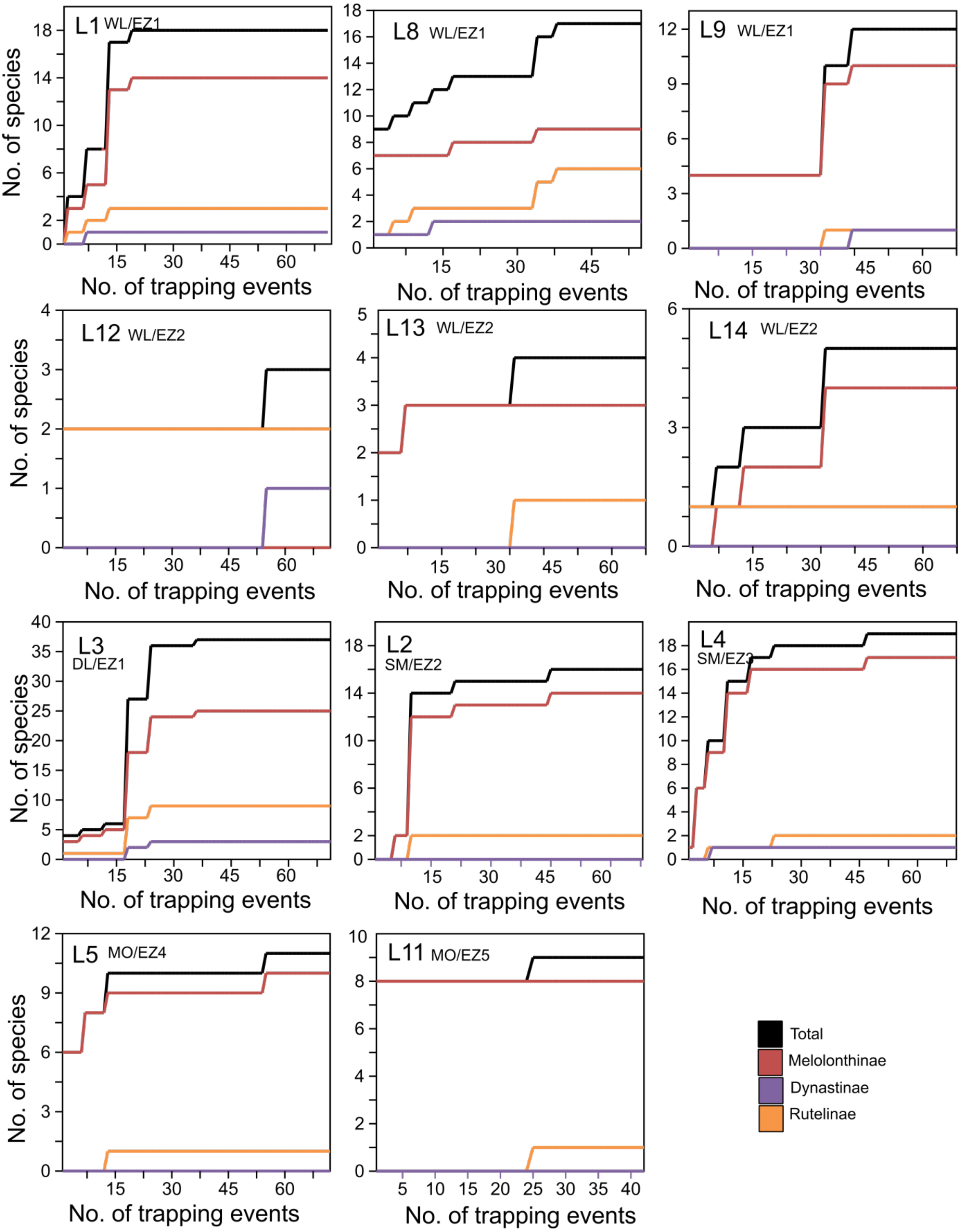

Figure S3: Species accumulation curves for each sampling locality for total species and subfamily level.

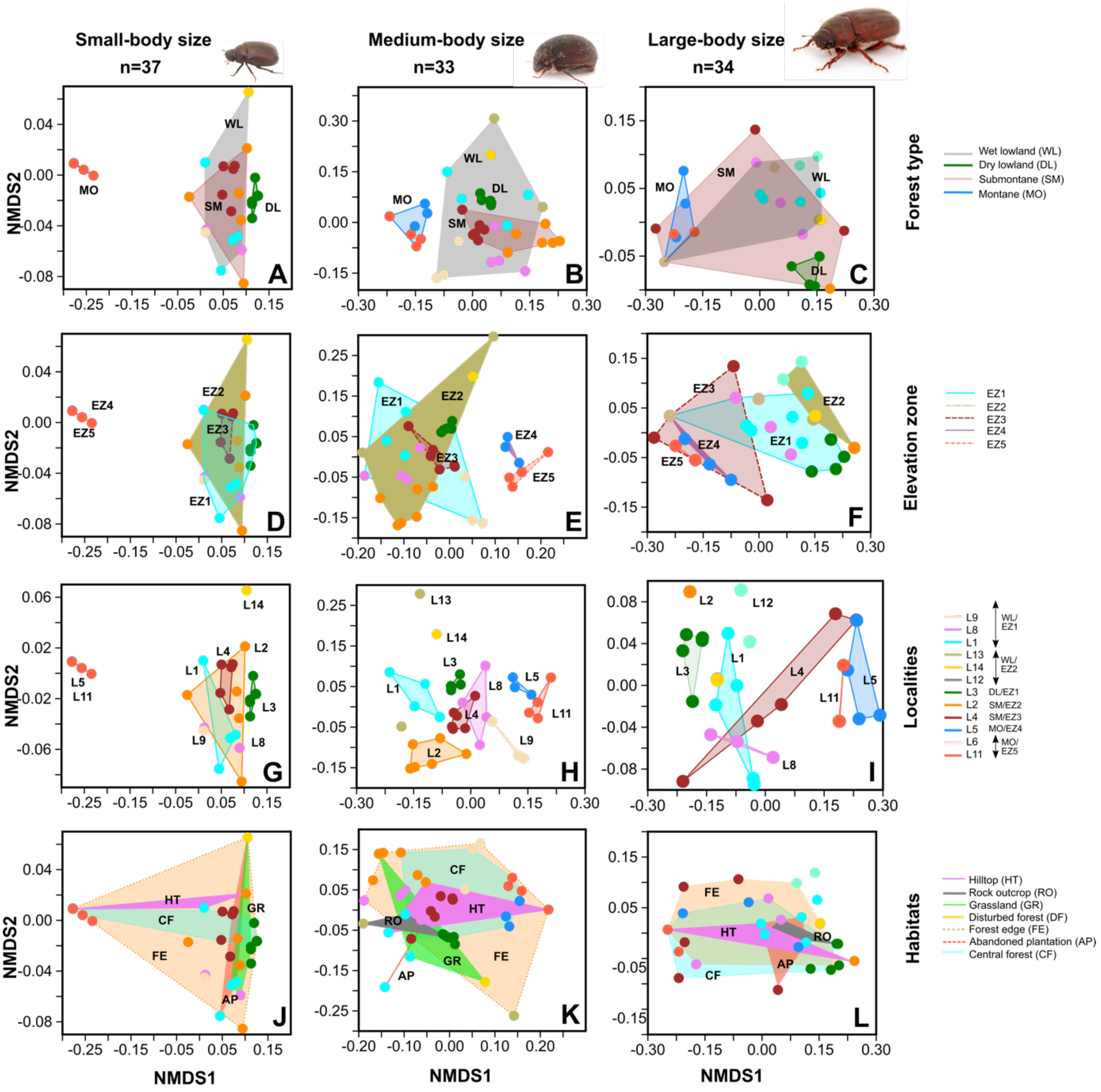

Figure S4: NMDS analyses assemblages separated by body size classes (percentile based) and different spatial and eco-spatial partitions; forest types (**A-C**), elevation zones (**D-F**), localities (**G-I**) and habitats (**J-L**). Partitions are enclosed by convex hulls. Multiple traps from one locality have the same colour and colours correspond to Figure S1.

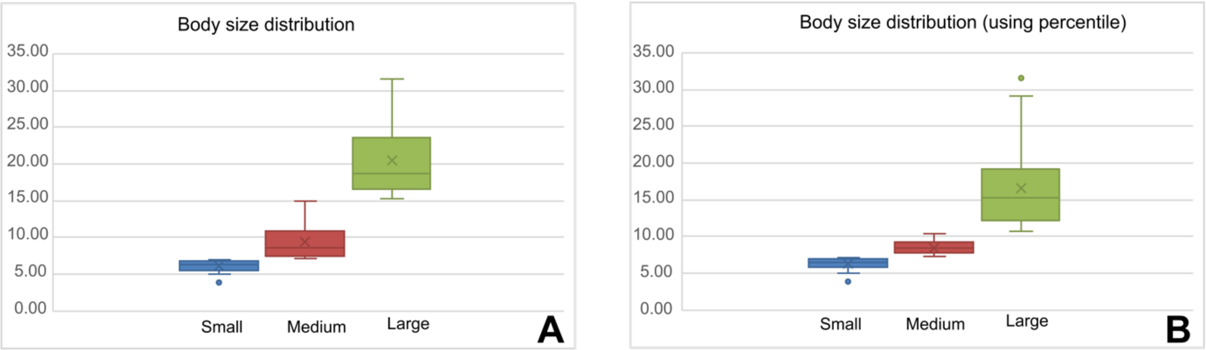

Figure S5: Body size distribution of specimens onto the two alternative size partition schemes (A - lineage (genus level) based; B – percentile based; with 33% of specimens assigned to each class).
