# Supplementary material for: Local stochastics and ecoclimatic situation shape phytophagous chafer assemblage composition
Source: Ecol Evol. 2023 May 11;13(5):e10091. doi: 10.1002/ece3.10091 (PMC10175718; doi:10.1002/ece3.10091)
Supplement: Supplementary file 2 — Data S2 [file ECE3-13-e10091-s001.pdf]

Table S1: Details of sampling sites, habitat types.

WL: wet lowland; DL: dry lowland; SM: sub-montane; MO: montane. EZ1: 0–500 m, EZ2: 501–1000 m, EZ3: 1001–1500 m, EZ4: 1501–2000 m, EZ5; 2001–2500 m. L: localities. A-F; trap IDs.

L	Site	Latitude	Longitude	FR	EZ	Habitat	Trap
L1	Aranayake	7.1507	80.4629	WL	EZ1	Abandon plantation	1B
L1	Aranayake	7.1505	80.4624	WL	EZ1	Grassland	1C
L1	Aranayake	7.1615	80.4639	WL	EZ1	Hilltop	1D
L1	Aranayake	7.1509	80.4629	WL	EZ1	Abandon Plantation	1A
L1	Aranayake	7.1583	80.4667	WL	EZ1	Abandon Plantation	1E
L1	Aranayake	7.1442	80.4503	WL	EZ1	Rock outcrop	1F
L11	Piduruthlagala	6.9896	80.7713	MO	EZ5	Central forest	11B
L11	Piduruthlagala	6.9988	80.7764	MO	EZ5	Central forest	11C
L11	Piduruthlagala	7.0003	80.7753	MO	EZ5	Central forest	11D
L11	Piduruthlagala	6.9785	80.7794	MO	EZ5	Forest Edge	11E
L11	Piduruthlagala	6.9787	80.7920	MO	EZ5	Forest Edge	11F
L11	Piduruthlagala	6.9830	80.7731	MO	EZ5	Hilltop	11A
L12	UdaPeradeniya	7.2480	80.6152	WL	EZ2	Disturbed forest	12B
L12	UdaPeradeniya	7.2487	80.6145	WL	EZ2	Disturbed forest	12C
L12	UdaPeradeniya	7.2475	80.6146	WL	EZ2	Disturbed forest	12D
L12	UdaPeradeniya	7.2506	80.6129	WL	EZ2	Forest Edge	12A
L12	UdaPeradeniya	7.2454	80.6140	WL	EZ2	Forest Edge	12F
L12	UdaPeradeniya	7.2470	80.6149	WL	EZ2	Grassland	12E
L13	Gannoruwa	7.2833	80.5982	WL	EZ2	Abandon plantation	13C
L13	Gannoruwa	7.2837	80.5983	WL	EZ2	Disturbed forest	13D
L13	Gannoruwa	7.2840	80.5983	WL	EZ2	Disturbed forest	13E
L13	Gannoruwa	7.2834	80.5990	WL	EZ2	Disturbed forest	13F
L13	Gannoruwa	7.2837	80.5987	WL	EZ2	Forest Edge	13B
L13	Gannoruwa	7.2834	80.5984	WL	EZ2	Rock outcrop	13A
L14	Udawattakele	7.2992	80.6425	WL	EZ2	Central forest	14D
L14	Udawattakele	7.2981	80.6500	WL	EZ2	Central forest	14F
L14	Udawattakele	7.3965	80.6540	WL	EZ2	Disturbed forest	14E
L14	Udawattakele	7.2973	80.6419	WL	EZ2	Forest Edge	14A
L14	Udawattakele	7.2967	80.6424	WL	EZ2	Forest valley	14B
L14	Udawattakele	7.2959	80.6422	WL	EZ2	Grassland	14C
L2	Riverston	7.5498	80.7521	SM	EZ2	Central forest	2C
L2	Riverston	7.5364	80.7723	SM	EZ2	Forest Edge	2A
L2	Riverston	7.5383	80.7500	SM	EZ2	Forest Edge	2E
L2	Riverston	7.5383	80.7511	SM	EZ2	Forest Edge	2F
L2	Riverston	7.5491	80.7539	SM	EZ2	Grassland	2B
L2	Riverston	7.5522	80.7529	SM	EZ2	Hilltop	2D
L3	Dambulla	7.8577	80.6747	DL	EZ1	Central forest	3B
L3	Dambulla	7.8580	80.6755	DL	EZ1	Central forest	3C
L3	Dambulla	7.8591	80.6759	DL	EZ1	Central forest	3F
L3	Dambulla	7.8590	80.6753	DL	EZ1	Forest Edge	3D
L3	Dambulla	7.8582	80.6751	DL	EZ1	Grassland	3E
L3	Dambulla	7.8578	80.6739	DL	EZ1	Rock outcrop	3A
L4	Deenston	7.3310	80.8593	SM	EZ3	Abandon plantation	4A
L4	Deenston	7.3350	80.8597	SM	EZ3	Central forest	4D
L4	Deenston	7.3362	80.8591	SM	EZ3	Central forest	4E
L4	Deenston	7.3389	80.8510	SM	EZ3	Central forest	4F
L4	Deenston	7.3316	80.8611	SM	EZ3	Forest Edge	4B
L4	Deenston	7.3308	80.8620	SM	EZ3	Forest Edge	4C
L5	NuwaraEliya	6.9113	80.7948	MO	EZ4	Central forest	5B
L5	NuwaraEliya	6.9109	80.7943	MO	EZ4	Central forest	5C
L5	NuwaraEliya	6.9307	80.8134	MO	EZ4	Central forest	5E

L5	NuwaraEliya	6.9300	80.8136	MO	EZ4	Central forest	5F
L5	NuwaraEliya	6.9118	80.7949	MO	EZ4	Forest Edge	5A
L5	NuwaraEliya	6.9305	80.8136	MO	EZ4	Forest Edge	5D
L8	Hiyare	6.0564	80.3170	WL	EZ1	Central forest	8C
L8	Hiyare	6. 070	80.3161	WL	EZ1	Central forest	8E
L8	Hiyare	6. 092	80.3320	WL	EZ1	Central forest	8F
L8	Hiyare	6.0596	80.3150	WL	EZ1	Forest Edge	8A
L8	Hiyare	6. 057	80.3151	WL	EZ1	Forest Edge	8B
L8	Hiyare	6.0587	80.3154	WL	EZ1	Grassland	8D
L9	Kottawa	6.0971	80.3167	WL	EZ1	Central forest	9A
L9	Kottawa	6.0981	80.3161	WL	EZ1	Central forest	9C
L9	Kottawa	6.1000	80.3086	WL	EZ1	Central forest	9D
L9	Kottawa	6.1345	80.3000	WL	EZ1	Central forest	9E
L9	Kottawa	6.0980	80.3167	WL	EZ1	Forest Edge	9B
L9	Kottawa	6.1367	80.3456	WL	EZ1	Forest valley	9F

Table S2: Details of species, recorded localities and their presence.

WL: wet lowland; DL: dry lowland; SM: sub-montane; MO: montane. EZ1: 0–500 m, EZ2: 501–1000 m, EZ3: 1001–1500 m, EZ4: 1501–2000 m, EZ5; 2001–2500 m. L: localities.

**Location**	**L1**	**L8**	**L9**	**L12**	**L13**	**L14**	**L3**	**L2**	**L4**	**L5**	**L11**	**L6**
**Forest/ Elevation zone**	**WL/ EZ1**	**WL/ EZ1**	**WL/ EZ1**	**WL/ EZ2**	**WL/ EZ2**	**WL/ EZ2**	**DL/ EZ1**	**SM/ EZ2**	**SM/ EZ3**	**MO/ EZ4**	**MO/ EZ5**	**MO/ EZ5**
**Morphospecies**												
Adoretus sp1	1	0	0	0	0	0	0	0	0	0	0	0
Adoretus sp10	0	0	0	0	0	0	1	0	0	0	0	0
Adoretus sp11	0	0	0	0	0	0	1	0	0	0	0	0
Adoretus sp12	0	0	0	0	0	0	1	0	0	0	0	0
Adoretus sp2	0	0	0	0	0	0	1	1	0	0	0	0
Adoretus sp3	0	1	0	0	0	0	1	1	0	0	0	0
Adoretus sp4	0	0	0	0	0	0	0	0	0	1	1	0
Adoretus sp5	0	0	0	0	0	0	0	0	1	0	0	0
Adoretus sp6	0	0	0	0	0	1	0	0	0	0	0	0
Adoretus sp7	0	1	1	0	0	0	0	0	0	0	0	0
Adoretus sp8	0	0	0	1	0	0	0	0	0	0	0	0
Adoretus sp9	0	0	0	0	0	0	1	0	0	0	0	0
Anomala sp1	1	1	0	0	1	0	1	0	0	0	0	0
Anomala sp2	1	0	0	1	0	0	0	0	0	0	0	0
Anomala sp3	0	0	0	0	0	0	1	0	0	0	0	0
Anomala sp4	0	0	0	0	0	0	1	0	0	0	0	0
Apogonia comosa	0	0	0	0	0	0	1	0	0	0	0	0
A. coriacea	0	0	0	0	0	0	0	0	1	1	1	0
A. glabrilinea	1	1	1	0	0	0	0	1	1	0	0	0
A. ludificans	0	0	0	0	0	0	0	1	1	0	0	0
A. nietneri	0	0	1	0	0	0	0	0	0	0	0	0
A. solida sp3	1	0	0	0	0	0	0	0	0	0	0	0
A. solida sp4	0	0	0	0	0	0	1	0	0	0	0	0
Apogonia sp1	1	1	0	0	0	0	1	0	1	0	0	0
Apogonia sp10	0	0	0	0	0	0	0	0	1	0	1	0
Apogonia sp12	0	0	0	0	0	0	0	0	1	0	0	0
Apogonia sp14	0	0	0	0	0	0	1	0	0	0	0	0
Apogonia sp15	0	0	0	0	0	0	1	0	0	0	0	0
Apogonia sp5	0	0	0	0	0	0	1	0	0	0	0	0
Apogonia sp7	0	0	0	0	0	0	1	0	0	0	0	0
Apogonia sp8	0	0	1	0	0	0	0	0	0	1	1	0
Brahmina sp1	0	0	0	0	0	0	0	0	0	1	0	0
Eophileurus sp2	0	0	0	0	0	0	1	0	0	0	0	0
Eophileurus sp6	0	0	0	0	0	0	0	0	1	0	0	0
Holotrichia sp1	1	0	0	0	0	0	0	0	0	0	0	0
Holotrichia sp2	1	0	0	0	0	0	0	0	0	0	0	0
Holotrichia sp3	0	0	0	0	0	0	1	0	0	0	0	0
Holotrichia sp4	0	0	0	0	0	1	0	0	0	0	0	0
Holotrichia sp5	0	1	1	0	0	0	0	0	1	0	0	0
Holotrichia sp6	0	0	0	0	0	0	0	0	1	0	0	0
Holotrichia sp7	1	0	0	0	0	0	0	0	0	0	0	0
Leucopholis sp1	1	0	0	0	0	0	0	0	0	0	0	0
Leucopholis sp2	1	1	1	0	0	0	0	0	0	0	0	0
Maladera anderssoni	0	0	0	0	0	0	0	0	1	0	0	0
M. badullana	0	0	0	0	0	0	0	0	0	1	1	0
M. bandarawelana	0	0	0	0	0	0	0	1	0	0	0	0
M. breviatella	0	0	0	0	0	1	1	0	0	0	0	0
M. calcarata	0	0	0	0	0	0	1	0	0	0	0	0
M. cervicornis	0	0	0	0	0	0	0	1	0	0	0	0

M. cinnaberina	1	0	0	0	0	0	0	0	0	0	0	0
M. coxalis	1	0	0	0	0	0	1	0	0	0	0	0
M. dambullana	0	0	0	0	0	0	1	0	0	0	0	0
M. deenstana	0	0	0	0	0	0	0	0	1	0	0	0
M. dubia	0	0	0	0	0	0	0	0	0	1	1	0
M. fistulosa	0	0	0	0	0	0	0	0	0	1	0	0
M. galdaththana	1	0	0	0	1	0	0	1	0	0	0	0
M. haniel	0	0	0	0	0	0	0	0	1	0	0	0
M. heveli	0	0	0	0	0	0	1	0	0	0	0	0
M. hiyarensis	0	1	0	0	0	0	0	0	0	0	0	0
M. hortonensis	0	0	0	0	0	0	0	0	0	0	1	0
M. iuga	0	0	0	0	0	0	0	0	1	0	0	0
M. kandyensis	0	0	0	0	1	1	0	0	0	0	0	0
M. karunaratnae	0	0	0	0	0	0	1	1	0	0	0	0
M. kishi	0	0	0	0	0	0	0	0	1	0	0	0
M. laterita	0	0	0	0	0	0	0	1	0	0	0	0
M. lindulana	0	0	0	0	0	0	0	0	1	0	0	0
M. mollis	0	0	0	0	0	0	1	0	0	0	0	0
M. padaviyaensis	0	0	0	0	0	0	1	0	0	0	0	0
M. pubescens	1	0	0	0	0	0	0	0	0	0	0	0
M. rotundata	1	0	0	0	0	0	0	0	0	0	0	0
M. rufocuprea	1	1	0	0	0	0	1	1	1	0	0	0
M. setosa	0	0	0	0	0	0	1	0	0	0	0	0
M. tricuspidata	0	0	0	0	0	0	1	0	0	0	0	0
M. weligamana	0	0	0	0	0	0	0	0	0	1	0	0
M. windy	0	0	0	0	0	0	0	0	1	0	0	0
Maladera sp	0	0	0	0	0	0	1	0	0	0	0	0
Mimela sp1	0	1	0	0	0	0	0	0	0	0	0	0
Neoserica dharmapriyai	1	0	0	0	0	0	0	0	0	0	0	0
N. pophami	0	0	0	0	0	0	1	0	0	0	0	0
N. sexfoliata	0	0	0	0	0	0	1	0	0	0	0	0
Orphnus sp1	1	0	0	0	0	0	0	0	0	0	0	0
Orphnus sp1	0	0	0	0	0	0	1	0	0	0	0	0
*Orphnus*sp	0	0	0	0	0	0	1	0	0	0	0	0
Oryctes sp7	0	1	0	0	0	0	0	0	0	0	0	0
Parastasiasp	0	1	0	0	0	0	0	0	1	0	0	0
Perisericasp	0	0	0	0	0	0	0	0	1	0	0	0
Phyllognathus sp8	1	0	0	0	0	0	0	0	0	0	0	0
Selaserica athukoralai	0	0	0	0	0	0	0	1	0	0	0	0
Sel. convexiuscula	0	0	1	0	0	0	0	0	0	0	0	0
Sel. fabriziae	0	0	1	0	0	0	0	0	0	0	0	0
Sel. impexa	0	1	0	0	0	0	0	0	0	0	0	0
Sel. maculicauda	0	0	0	0	0	0	0	0	0	0	0	1
Sel. nitida	0	0	0	0	0	0	0	0	0	1	1	0
Sel. nuwarana	0	0	0	0	0	0	0	0	0	1	0	0
Sel. praetexta	0	0	0	0	1	1	0	1	0	0	0	0
Sel. pusilla	0	0	0	0	0	0	1	1	0	0	0	0
Sel. sororinitida	0	0	0	0	0	0	0	0	1	0	0	0
Serica fusa	0	0	0	0	0	0	0	0	0	1	1	0
Serica lurida	0	0	0	0	0	0	0	1	1	0	0	0
Sophrops sp1	1	1	0	0	0	0	1	0	0	0	0	0
Sophrops sp2	0	1	1	0	0	0	1	1	1	0	0	0
Sophrops sp3	0	0	1	0	0	0	0	0	0	0	0	0
Sophrops sp4	0	0	0	0	0	0	1	1	0	0	0	0
Sophrops sp5	0	0	0	0	0	0	1	0	0	0	0	0
Xylotrupes sp5	1	1	1	1	0	0	0	0	0	0	0	0

Table S3: Individuals, observed species richness (Sobs) and percentages of inventory completeness and diversity indices of scarab beetles in all sampling locations. WL: wet lowland; DL: dry lowland; SM: sub-montane; MO: montane. EZ1: 0–500 m, EZ2: 501–1000 m, EZ3: 1001–1500 m, EZ4: 1501–2000 m, EZ5; 2001–2500 m. L: localities.

Location<br>Forest/<br>Elevation zone	L1<br>WL/<br>EZ1	L8<br>WL/<br>EZ1	L9<br>WL/<br>EZ1	L12<br>WL/<br>EZ2	L13<br>WL/<br>EZ2	L14<br>WL/<br>EZ2	L3<br>DL/<br>EZ1	L2<br>SM/<br>EZ2	L4<br>SM/<br>EZ3	L5<br>MO/<br>EZ4	L11<br>MO/<br>EZ5
**No. of species**	19	16	12	3	4	5	38	16	19	12	9
**No. of specimens**	231	55	42	9	28	8	1837	184	281	183	116
**Choa1**	21.5	19	24	3	4.5	11	39.11	18	19.14	12	10
**% Completeness**	88.37	84.21	50.00	100.00	88.89	45.45	97.16	88.89	99.27	100.00	90.00
**Simpson_1-D**	0.50	0.84	0.52	0.62	0.28	0.69	0.88	0.72	0.82	0.84	0.80
**Shannon_H**	1.41	2.28	1.37	1.03	0.61	1.39	2.54	1.73	2.06	2.06	1.81
**Evenness_e^H/S**	0.22	0.61	0.33	0.93	0.46	0.80	0.33	0.35	0.41	0.65	0.68
**Hill numbers q=1**	4.09	9.79	3.94	2.80	1.83	4.00	10.02	5.63	7.81	7.82	6.13
**Hill numbers q=2**	2.01	6.21	2.10	2.63	1.40	3.20	7.41	3.53	5.43	6.43	4.89

Table S4: Pearson correlation between the similarity of chafer assemblage sorted for body size and for separate lineages with geographic distance. Significant correlation (R) coefficients ($p < 0.05$) are printed in bold.

**Partition**		**R**	**p**
**Body size**	small	**-0.344**	0.02
	medium	0.112	0.46
	large	-0.023	0.92
**Lineage**	Dynastinae	0.291	0.19
	Rutelinae	-0.110	0.42
	Sericini	0.239	0.07
	Melolonthinae	0.178	0.19

Table S5: Kruskal-Wallis test for species turnover in localities between four field campaigns (first row). Significant seasonal species turnover ($p < 0.05$) is printed in bold. Percentage seasonal species turnover in localities between four field campaigns (second row).

	**L1**	**L2**	**L3**	**L4**	**L5**	**L8**	**L9**	**L11**	**L12**	**L13**	**L14**
**p**	**1.75E-03**	**0.005519**	**3.32E-06**	0.4761	0.4241	0.06445	0.004122	0.3914	0.7014	0.6516	1
**Species turnover %**	59.73	61.47	48.95	44.24	39.96	48.15	47.39	19.23	48.89	30.00	33.33

Table S6: Similarity (Jaccard measure) in species composition among campaigns for total assemblage and assemblage sorted for body size and lineages.

Total assemblage	**2019-I**	**2019-II**	**2020-I**	**2020-II**	***Sercini***	**2019-I**	**2019-II**	**2020-I**	**2020-II**
**2019-I**	x				**2019-I**	x			
**2019-II**	0.31	x			**2019-II**	0.34	x		
**2020-I**	0.23	0.17	x		**2020-I**	0.18	0.22	x	
**2020-II**	**0.49**	0.44	0.25	x	**2020-II**	**0.55**	0.44	0.32	x

Large	**2019-I**	**2019-II**	**2020-I**	**2020-II**	***Melolonthinae***	**2019-I**	**2019-II**	**2020-I**	**2020-II**
**2019-I**	x				**2019-I**	x			
**2019-II**	0.25	x			**2019-II**	0.34	x		
**2020-I**	0.19	0.15	x		**2020-I**	0.32	0.17	x	
**2020-II**	0.29	**0.40**	0.18	x	**2020-II**	0.45	**0.52**	0.20	x

Medium	**2019-I**	**2019-II**	**2020-I**	**2020-II**	***Dynastinae***	**2019-I**	**2019-II**	**2020-I**	**2020-II**
**2019-I**	x				**2019-I**	x			
**2019-II**	0.29	x			**2019-II**	0.17	x		
**2020-I**	0.31	0.24	x		**2020-I**	0.20	**0.25**	x	
**2020-II**	**0.49**	0.39	0.33	x	**2020-II**	0.20	**0.25**	0	x

Small	**2019-I**	**2019-II**	**2020-I**	**2020-II**	***Rutelinae***	**2019-I**	**2019-II**	**2020-I**	**2020-II**
**2019-I**	x				**2019-I**	x			
**2019-II**	0.31	x			**2019-II**	0.13	x		
**2020-I**	0.08	0.04	x		**2020-I**	0.29	0.06	x	
**2020-II**	0.50	**0.52**	0.13	x	**2020-II**	0.22	**0.40**	0.20	x
